# In Lysinuric Protein Intolerance system y^+^L activity is defective in monocytes and in GM-CSF-differentiated macrophages

**DOI:** 10.1186/1750-1172-5-32

**Published:** 2010-11-26

**Authors:** Amelia Barilli, Bianca Maria Rotoli, Rossana Visigalli, Ovidio Bussolati, Gian C Gazzola, Zamir Kadija, Giuseppe Rodi, Francesca Mariani, Maria Lorena Ruzza, Maurizio Luisetti, Valeria Dall'Asta

**Affiliations:** 1 Dipartimento di Medicina Sperimentale, Sezione di Patologia Generale e Clinica, Università degli Studi di Parma, Parma, Italy; 2 Clinica Malattie Apparato Respiratorio Fondazione IRCCS Policlinico San Matteo, Università di Pavia, Pavia, Italy; 3 Servizio Rianimazione 1, Fondazione IRCCS Policlinico San Matteo, Università di Pavia, Pavia, Italy; 4 Dipartimento Materno Infantile, Ospedale San Carlo Borromeo, Milano, Italy

## Abstract

**Background:**

In the recessive aminoaciduria Lysinuric Protein Intolerance (LPI), mutations of *SLC7A7*/y+LAT1 impair system y^+^L transport activity for cationic amino acids. A severe complication of LPI is a form of Pulmonary Alveolar Proteinosis (PAP), in which alveolar spaces are filled with lipoproteinaceous material because of the impaired surfactant clearance by resident macrophages. The pathogenesis of LPI-associated PAP remains still obscure. The present study investigates for the first time the expression and function of y+LAT1 in monocytes and macrophages isolated from a patient affected by LPI-associated PAP. A comparison with mesenchymal cells from the same subject has been also performed.

**Methods:**

Monocytes from peripheral blood were isolated from a 21-year-old patient with LPI. Alveolar macrophages and fibroblastic-like mesenchymal cells were obtained from a whole lung lavage (WLL) performed on the same patient. System y^+^L activity was determined measuring the 1-min uptake of [^3^H]-arginine under discriminating conditions. Gene expression was evaluated through qRT-PCR.

**Results:**

We have found that: 1) system y^+^L activity is markedly lowered in monocytes and alveolar macrophages from the LPI patient, because of the prevailing expression of *SLC7A7*/y+LAT1 in these cells; 2) on the contrary, fibroblasts isolated from the same patient do not display the transport defect due to compensation by the *SLC7A6*/y+LAT2 isoform; 3) in both normal and LPI monocytes, GM-CSF induces the expression of *SLC7A7*, suggesting that the gene is a target of the cytokine; 4) GM-CSF-induced differentiation of LPI monocytes is comparable to that of normal cells, demonstrating that GM-CSF signalling is unaltered; 5) general and respiratory conditions of the patient, along with PAP-associated parameters, markedly improved after GM-CSF therapy through aerosolization.

**Conclusions:**

Monocytes and macrophages, but not fibroblasts, derived from a LPI patient clearly display the defect in system y^+^L-mediated arginine transport. The different transport phenotypes are referable to the relative levels of expression of *SLC7A7 *and *SLC7A6*. Moreover, the expression of *SLC7A7 *is regulated by GM-CSF in monocytes, pointing to a role of y+LAT1 in the pathogenesis of LPI associated PAP.

## Background

Lysinuric Protein Intolerance (LPI, MIM 222700) is an autosomic, recessive, hyperdibasic aminoaciduria caused by defective cationic amino acid (CAA; L-arginine, L-lysine, L-ornithine) transport at the basolateral membrane of epithelial cells in the intestine and kidney [1]. The defect affects transport system y^+^L, a member of the large group of heterodimeric amino acid transporters formed by a light subunit, which may be either y+LAT1 (encoded by the *SLC7A7 *gene) or y+LAT2 (*SLC7A6 *gene), and a glycoprotein (4F2 hc/CD98 hc) that is necessary for the correct expression of the transporter in the plasma membrane [2]. Two groups independently identified *SLC7A7 *as the gene mutated in LPI [3,4]. Because of the transport defect, LPI patients have high renal clearance and low intestinal absorption of CAA and, as a consequence, their CAA plasma levels are usually low [5].

The clinical presentation of the disease is characterized by hyperammonemia, gastrointestinal symptoms, failure to thrive, renal disease, and osteoporosis [5]. Additional features, which are not obviously related to the transport derangement, include hematopoietic abnormalities, chronic renal disease, and lung involvement [6]. Pulmonary manifestations are variable and range from subclinical interstitial lung disease to severe complications, eventually leading to fatal Pulmonary Alveolar Proteinosis [7-9].

Pulmonary Alveolar Proteinosis (PAP) is a rare disorder in which alveolar spaces of the lungs are excessively filled with lipoproteinaceous material (surfactant) leading to progressive respiratory insufficiency [10]. Alveolar macrophages (AM) appear foamy, lipid-filled because of the impaired surfactant clearance in these cells. Multiple clinical forms of PAP have been described according to the presumed aetiology [11]: (1) idiopathic (primary) PAP, the most common form diagnosed in adults, which may be either congenital (mutations of α or β chains of the receptor for GM-CSF) or acquired (autoimmune PAP with autoantibodies targeting GM-CSF); (2) secondary PAP, resulting from conditions in which AM function is suppressed, such as immunodeficiency states, hematologic malignancies, exposure to inorganic dusts (eg, silica), or pharmacologically induced. Among the secondary forms, LPI-associated PAP resembles clinically autoimmune PAP with accumulation of surfactant lipids and proteins in the airspaces and enlarged AM that contain numerous phospholipids inclusions [12].

Despite all the progresses in the elucidation of PAP pathogenesis [13], the molecular mechanisms underlying LPI-associated PAP remain enigmatic and the reason why *SLC7A7 *mutations lead to lung involvement in LPI patients is still obscure. A hypothesis is that PAP secondary to LPI might be due to a disorder of bone marrow-derived monocytes that differentiate into dysfunctional alveolar macrophages. Indeed, a LPI patient relapsed after heart-lung transplantation for severe, PAP-associated respiratory insufficiency and died of respiratory failure after a period of clinical remission [14]. This evidence suggests that circulating cells of the patient, such as monocytes/macrophages, colonize the lung after the transplant and reproduce the pathological condition. Consistently, we demonstrated high expression of *SLC7A7 *in human peripheral monocytes and in human alveolar macrophages (AM), as well as system y^+^L as the only functional transport activity for CAA in these cell models [15,16]. The high level of *SLC7A7 *expression found in these cells strengthens the hypothesis that y+LAT1 defect in macrophages plays an important role in the pathogenesis of LPI-associated PAP.

In the present study, we compare the activity of system y^+^L and the expression of CAA transporters in peripheral blood monocytes, AM and fibroblasts isolated from a young man affected by LPI, who developed PAP eleven years ago, and in control cells. The effect of GM-CSF on the differentiation of monocytes has been also addressed and found comparable in control and LPI cells, providing a rationale for GM-CSF therapy in LPI-associated PAP.

## Methods

### Isolation of the cells

#### Isolation of monocytes

Two different isolations of peripheral monocytes were performed from the LPI patient at a distance of 3 months. Nine normal healthy donors were used as controls. 20 ml of heparinized blood, diluted with PBS, were layered on Lympholyte gradient medium (Cedarlane Laboratories, Celbio, Italy) and centrifuged at 800 *g *for 15 min at 20°C. Peripheral blood mononuclear cells (PBMC) from the interface were collected and washed in RPMI medium by centrifugation at 200 *g *for 7 min at 20°C. Cells were suspended in RPMI containing 10% endotoxin-free Fetal Bovine Serum (FBS) and seeded on plastic-ware appropriate for the various determinations. After a 90-min incubation at 37°C in an atmosphere at 5% CO_2_, non-adherent cells were removed with vigorous washes in pre-warmed RPMI medium. Adherent monocytes were employed immediately, for the characterization of arginine transport and the analysis of CAA transporters expression, or cultured to obtain monocyte-derived macrophages (MDM). These cells were obtained by culturing monocytes for 5 days in RPMI with 10% FBS, supplemented with 10 ng/ml of recombinant human Granulocyte Mϕ-Colony Stimulating Factor (rGM-CSF).

#### Isolation of alveolar macrophages

Alveolar macrophages (AM) were isolated from Whole Lung Lavage (WLL) of the LPI patient. The lavage fluid obtained from the clinical procedure was centrifuged for 10 minutes at 400 *g*. The supernatant was discarded, while the pellet, containing large amounts of amorphous lipoproteinaceous material, was diluted with RPMI and seeded in 24-well trays or 3-cm diameter dishes (Falcon). After 2 h, the excess fluid was discarded through several washes with medium and adherent cells, mainly foamy macrophages, were used immediately for the characterization of arginine transport and the analysis of CAA transporters expression.

AM from normal healthy subjects (n = 3) were obtained from bronchoalveolar lavage (BAL) after written informed consent. Bronchoalveolar lavage (BAL) and AM isolation were performed as previously described [16].

#### Isolation of fibroblastic-like mesenchymal cells

**Figure 1:**
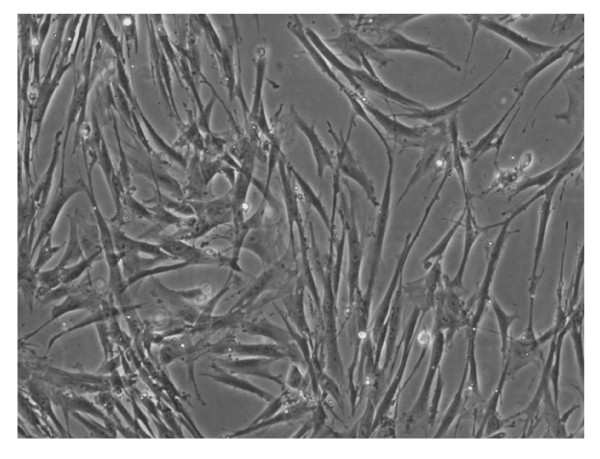
**Phase contrast image of fibroblast-like mesenchymal cells obtained from the LPI subject**. Cells were isolated from WLL fluid, as described in Methods. × 100

The lavage fluid obtained from WLL was processed as described above, with the sole difference that, after dilution with RPMI, the pellet was seeded in 10-cm diameter dishes (Falcon) and incubated at 37°C, 5% CO_2_. for 24 h. After this period, the incubation medium was discarded and, after several washes with RPMI, substituted with fresh complete RPMI medium. Adherent cells, which appeared mostly as foamy macrophages, were left at 37°C, 5% CO_2 _for one week. At this time, a heterogeneous population consisting primarily of macrophages and fibroblast-like cells was evident. After 2-3 passages of routinely culture growing, the culture appeared homogeneously composed by fibroblastic cells (Figure 1). Normal human fibroblasts were isolated from skin biopsies of 3 healthy donors as already described [17]. Normal and LPI fibroblasts were routinely cultured in RPMI supplemented with 10% FBS, streptomycin (100 μg/ml) and penicillin (100 U/ml) in a humidified atmosphere of 5% CO_2 _in air.

### L-Arginine Influx

For transport studies, cells were seeded on 24-well (AM) or 96-well (monocytes and fibroblasts) dishes. Cells, washed once with a modified bicarbonate-free Earle's Balanced Salt Solution (EBSS), buffered at pH 7.4 with 20 mM Tris/HCl, were incubated for 1 min in the same solution containing [^3^H]-arginine (4 μCi/ml, 50 μM). In this interval of time arginine uptake approached linearity (results not shown). The experiment was terminated by two rapid washes (< 10 sec) in ice-cold PBS and cell monolayers were extracted in ethanol. The radioactivity in cell extracts was determined with Microbeta Trilux (Wallac). Extracted cells were then dissolved with 0.5% sodium deoxycholate in 1 M NaOH and protein content was determined directly in the well using a modified Lowry procedure, as previously described [15]. Arginine influx is expressed as nmol/mg of protein/min.

### qRT-Polymerase Chain Reaction

1 μg of total RNA, isolated with GenElute™ Mammalian Total RNA Miniprep Kit (Sigma, Milano, Italy), was reverse transcribed and 40 ng of cDNA were amplified as described previously [18]. The following forward and reverse primers (5 pmol each) were used: 5' GAA GGA GGA GCA TCA GAC CA 3' and 5' CCC AGT TCC GCA TAA CAA AG 3' for y^+^LAT1/*SLC7A7*; 5' CTT TCT ACT TCA TGG GTG TTT ACC TG 3' and 5' ATC CTG AGT CTC CTA TAG CTT ACC AA 3' for y^+^LAT2/*SLC7A6*; 5' TTT GAT GCT CGC TCA ATG ACA 3' and 5' CTT GAA GGG AAG GGC TGT TTT 3' for CD204; 5' CTG CTG GAT TGA CAA TAT CAG GC 3' and 5' CCC GTT TTC ATT TGG GGC TC 3' for LPLA2; 5' GAC AGG AAA GAC AAC AGA CAA ATC 3' and 5' TGT GTA AAT GAT CTC GTG GACTC 3' for PPARγ; 5' TGC CTC CAG TAC CCA TCC C 3' and 5' CCA CCC ACC AGA TGC TGT C 3' for PU.1; 5' GCA GCC ATC AGG TAA GCC AAG 3' and 5' AGC GGA CCC TCA GAA GAA AGC 3' for *RPL15*. Primers for *SLC7A7 *were designed so as to elude the known mutation site.

The expression of the gene of interest was normalized to that of the housekeeping gene *RPL15*. *SLC7A6*, *SLC7A7 *and *RPL15 *expression levels are given as numbers of mRNA molecules [19]. *CD204*, *LPLA2*, *PPARγ *and *PU.1 *are indexed to the housekeeping gene using the formula [16]:

(1) $$\text{1},000*{\text{2}}^{\Delta \text{Ct}}$$

where ΔCt = Ct_*RPL15 *_- Ct_proband gene_.

### Statistics

Student *t *test for unpaired data was used for statistical analysis of differences among treatments. Differences were considered significant when *p *was less than 0.05.

### Materials

Endotoxin-free fetal bovine serum (FBS), RPMI 1640 and Lympholyte (Cedarlane Laboratories) were purchased from EuroClone (Milan, Italy). [L-2,3,4-^3^H]Arginine (45-70 Ci/mmol) was obtained from Perkin-Elmer (Monza, Italy). rGM-CSF for in vitro experiments (ReliaTech) was purchased from TebuBio (Milan, Italy). Sigma-Aldrich (Milan, Italy) was the source of all the other chemicals.

## Results

### Case history

The patient is an Italian male, currently aged 21. Only one mutant allele of *SLC7A7 *gene was identified in the patient: this mutation, p.M50K (c.149T > A), is located in the TM domain I and causes the substitution of a highly conserved amino acid. The p.M50K mutation was inherited from the father [20]. Although several groups have tried to identify the mutation inherited by the mother, these attempts have been thus far unsuccessful.

**Figure 2:**
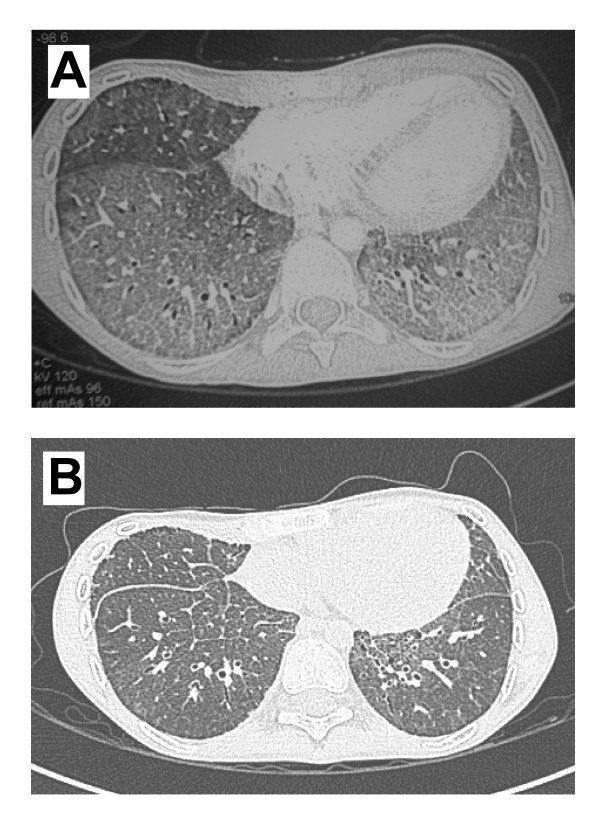
**Chest HRCT**. Panel A. Lung CT scan obtained before WLL (December 2008). Bilateral, diffuse ground glass of lower pulmonary lobes, with thickening of intra- and inter-lobular septa (crazy paving pattern). Panel B. Lung CT scan obtained after inhaled rGM-CSF trial (April 2010), showing a marked bilateral improvement in lower lobe involvement; nevertheless, lung density appears still increased.

The clinical history of the patient was already described in two papers [21,22]. Briefly, in the eighth month of life the baby was diagnosed as affected by LPI and, at the age of 15, a PAP was diagnosed based on Computed Tomography (CT) scan of a crazy paving pattern and of a mild restrictive ventilatory impairment. The patient was treated by whole lung lavage (WLL) according to the current standard of care. At a control chest CT scan performed 9 months after the WLL, the crazy paving pattern was almost totally resolved, but the lung density was slightly, diffusely increased with respect to normal lungs. After 4 years, the patient was newly admitted for fever and hypoxemia, and the lung CT scan revealed the relapse of PAP (Figure 2A). On January 2009 he underwent a WLL which resulted in an immediate improvement. The benefit this time was transient, and on March 2009 he was newly admitted to our Intensive Care Unit (ICU) because of severe respiratory failure. The third WLL, performed on March 2009, was complicated by an acute alveolar haemorrhage with acute anaemia, and the patient was treated with non-invasive ventilation and red blood cell transfusion. The persistence of respiratory failure suggested patient refractoriness to WLL and induced to consider a treatment with inhaled rGM-CSF (Sargramostim, Leukine, Bayer). The treatment was preceded by a bone marrow biopsy, aimed at excluding the presence of a latent haemophagocytic syndrome, a possible feature of LPI [23]. The rGM-CSF treatment was approved by the AIFA (Italian Agency For Drugs) and by the Ethic Committee of the San Matteo Hospital. The drug was nebulised daily with the PARI Boy (kindly provided by dr Arienti, Sapio Life Srl, Italy) at the dose of 250 μg daily for seven consecutive days, followed by 7 days off. This strategy was adopted so as to minimize possible side effects. rGM-CSF administration was repeated in 6 cycles, for a total of 42 vials (10,5 mg of rGM-CSF). The treatment was well tolerated and no side effects occurred. The respiratory conditions slightly improved and the oxygen supplementation required at rest gradually decreased from 6 L/minute to 1 L/minute. 3 weeks after the end of the rGM-CSF treatment, the patient experienced an acute chest pain and a severe dyspnea, and was admitted with a partial right pneumothorax, treated with aspiration. A second episode occurred at the end of August, and this time the complete pneumothorax was treated with a chest tube. Because of the difficult management of the pneumothorax, a second tube was placed, and this procedure was complicated by a severe pleural bleeding, requiring aspiration and red blood cell transfusion. In spite of these complications, the lung parenchyma progressively cleared, allowing the withdrawn of oxygen supplementation. The patient was discharged on September. In spite of three further episodes of partial apical pneumothorax, all spontaneously resolved with rest, the actual general and respiratory conditions of the patient are good and he is enjoying a satisfactory lifestyle. At the last visit (April 2010) oxygen saturation was 95% at rest and 87% after a 6 minutes walking test. At the same time, also the CT scan showed a marked improvement of PAP appearance, although the crazy paving pattern persisted (Figure 2B).

### Activity of system y^+^L in monocytes, alveolar macrophages and fibroblasts from the LPI patient

**Figure 3:**
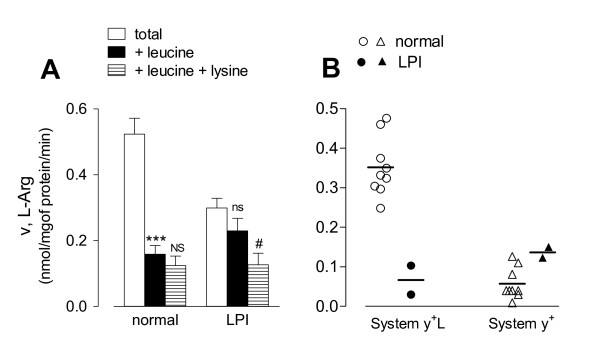
**Characterization of L-arginine influx in LPI monocytes**. Normal and LPI monocytes, isolated as described in Methods, were washed in EBSS. Panel A. Arginine uptake was assayed by 1 min incubation in EBSS supplemented with L-[^3^H]-arginine (50 μM; 4 μCi/ml) in the absence (total uptake) or in the presence of leucine (2 mM) or leucine + lysine (both 2 mM) as indicated. For normal cells, data are means ± SEM of 9 independent experiments (n = 9 normal donors), each performed in quadruplicate. For the LPI patient, data are means ± SD of 4 determinations obtained in a representative experiment, repeated twice with comparable results. *** p < 0.001 vs total; NS, Not Significant vs +Leucine; ns, not significant vs total; ^# ^p < 0.05 vs +Leucine. Panel B. Arginine transport values of each subject (n = 9 normal donors; n = 2 determinations in the LPI patient) were employed to calculate system y^+^L and system y^+ ^transport activity. System y^+^L: difference between total uptake and the uptake obtained in the presence of 2 mM leucine; system y^+^: difference between the influx measured in the presence of 2 mM leucine and that measured in the presence of 2 mM leucine + 2 mM lysine.

The transport of arginine (50 μM) in monocytes from normal subjects (n = 9) or from the LPI patient was determined under different experimental conditions selected to discriminate the relative contribution of systems y^+^L and y^+ ^[24]*(*Figure 3). An excess of leucine (2 mM) was used to inhibit system y^+^L transport activity, whereas leucine (2 mM) + lysine (2 mM) were employed to inhibit the whole saturable component of arginine transport. Therefore, the portion of arginine transport inhibited by leucine can be ascribed to system y^+^L activity, while the quote of uptake further inhibited by lysine corresponds to the contribution of system y^+^.

As expected for normal monocytes [15], leucine significantly inhibited arginine uptake and the addition of 2 mM lysine did not produce any further significant decrease of arginine transport (Panel A). LPI monocytes had a total arginine influx lower than normal monocytes and the presence of leucine during the uptake assay did not produce any significant inhibition of arginine transport (Panel A); in these cells, however, lysine caused a significant decrease of arginine uptake. The contributions of systems y^+^L and y^+ ^to arginine uptake was calculated from the transport values obtained from monocytes of the 9 normal subjects and of the patient (Panel B). System y^+^L activity was markedly lower in LPI monocytes (mean of two measurement 0.066 ± 0.051) than in cells from normal subjects (mean 0.35 ± 0.02; range 0.248 - 0.476). Conversely, system y^+ ^activity of LPI monocytes was in the upper range of transport values obtained with normal monocytes (mean of two measurement 0.135 ± 0.019 for LPI; mean 0.057 ± 0.013, range 0.009 - 0.126 for normal).

**Figure 4:**
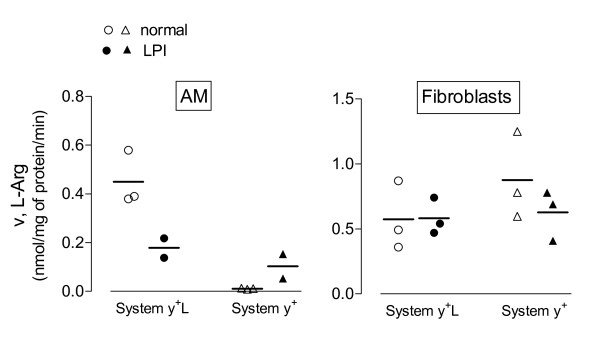
**System y^+^L and y^+ ^activities in alveolar macrophages (AM) and in fibroblasts obtained from the LPI subject**. Arginine uptake was assayed in AM or fibroblasts from different healthy donors (n = 3) or from the LPI patient (2 experiments on AM and 3 on fibroblasts) and system y^+^L and system y^+ ^transport activities were determined as described in Figure 3..

The same approach was adopted to measure arginine transport in alveolar macrophages (AM) and in fibroblast-like mesenchymal cells isolated from the LPI patient (see Methods). As demonstrated in a previous study [16], arginine transport in AM from healthy subjects is fully attributable to the activity of system y^+^L (Figure 4, Panel A). Similarly to what was observed in monocytes, system y^+^L activity was largely reduced in AM isolated from the LPI patient. Conversely, the activity of system y^+^L was readily detectable in LPI fibroblast-like mesenchymal cells and comparable to that observed in normal human fibroblasts. This result, besides confirming previous data obtained in a panel of LPI fibroblasts [25], suggests that mesenchymal cells do not express the transport defect referable to the mutation of *SLC7A7*.

### Expression of mRNA for system y^+^L transporters in monocytes, alveolar macrophages and fibroblasts of the LPI patient

**Figure 5:**
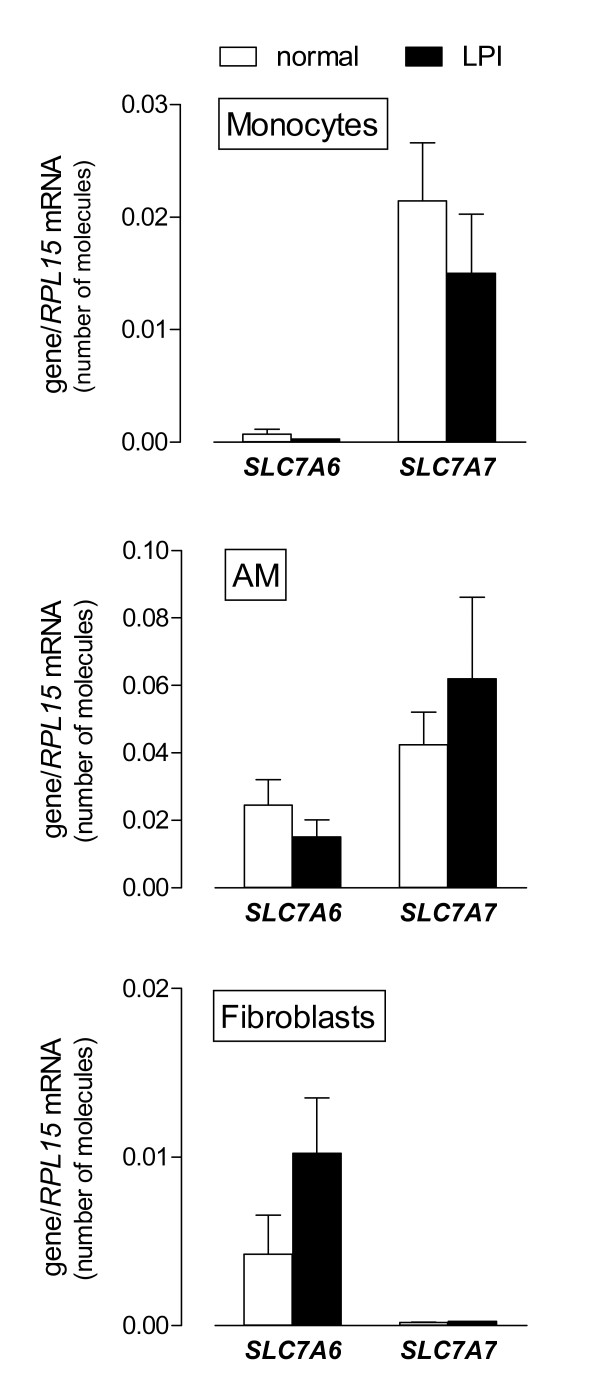
**Expression of *SLC7A6 *and *SLC7A7 *in monocytes, alveolar macrophages (AM) and fibroblasts obtained from the LPI subject**. After RNA extraction and reverse transcription, cDNA was employed as template for qPCR with *SLC7A6*/y+LAT2 and *SLC7A7*/y+LAT1 primers. mRNA level, normalized for *RPL15 *gene, is expressed as number of molecules (see Methods). For normal cells, data are means ± SEM of 9 (monocytes) or 3 (AM and fibroblasts) independent experiments. For the LPI patient, bars are means ± SD of 6 (monocytes and fibroblasts) or 4 (AM) determinations.

To assess if a different pattern of expression of *SLC7A7*/y+LAT1 and *SLC7A6*/y+LAT2 in LPI monocytes, macrophages and fibroblasts may justify the different transport phenotype of these cells, the expression of both genes was evaluated as number of mRNA molecules. This approach allows, indeed, the precise quantification of *SLC7A7 *and *SLC7A6 *mRNAs within a single cell model and quantitatively compares their expression among different cell types. As shown in Figure 5, *SLC7A7 *mRNA was highly expressed in monocytes and even more in AM, both from normal donors and from the LPI patient, while it appears extremely low in fibroblasts. On the contrary, *SLC7A6 *gene displayed a well evident expression in AM and fibroblasts, both from controls and LPI, and a very low expression in monocytes. The comparison between *SLC7A7 *and *SLC7A6 *expression levels indicated that in normal monocytes *SLC7A7 *mRNA was 30-fold more expressed than *SLC7A6 *(0.021 ± 0.005 molecules *SLC7A7*/*RPL15 *vs 0.0007 ± 0.00004 molecules *SLC7A6*/*RPL15*). On the contrary, in mesenchymal cells *SLC7A6 *mRNAs were about 20-fold more numerous than *SLC7A7 *mRNAs (0.004 ± 0.002 molecules *SLC7A6*/*RPL15 *vs 0.00019 ± 0.00002 molecules *SLC7A7*/*RPL15*). Comparable conclusions were reached for LPI cells. These data suggest that in LPI monocytes and AM the mutation in *SLC7A7 *cannot be complemented by the low relative expression of *SLC7A6*. On the contrary, LPI fibroblasts appear functionally comparable to normal cells because of the large excess of *SLC7A6*/y+LAT2..

### GM-CSF treatment in normal and LPI monocytes

**Figure 6:**
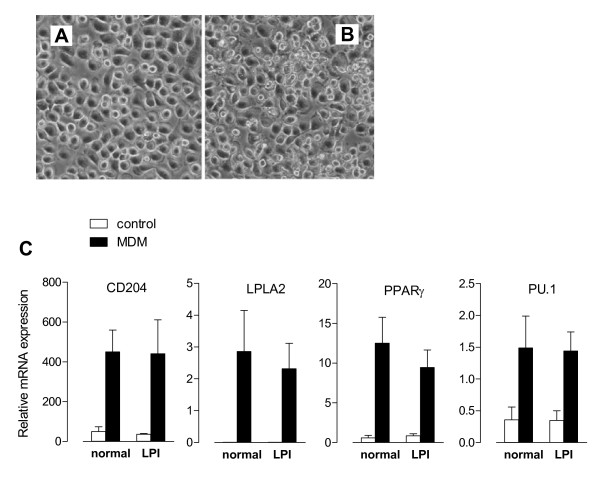
**Effect of GM-CSF on the differentiation of LPI monocytes to macrophages**. Normal and LPI monocytes were differentiated to Monocyte-Derived Marcophages (MDM) by 5 d incubation in RPMI supplemented with rGM-CSF (10 ng/ml). Panels A and B. Phase contrast images of normal (A) and LPI (B) MDM. × 100. Panel C. Induction of macrophage differentiation markers in normal and LPI MDM. In freshly isolated (control) or rGM-CSF-treated (MDM) monocytes RNA was extracted and cDNA employed as template for qPCR with primers for the indicated genes. The values are normalized to that of RPL (see Eq 1 in Methods). For normal cells, data are means ± SEM of 5 independent experiments (n = 5 normal donors). For the LPI patient, data are means ± SD of 4 determinations.

In order to assess if LPI monocytes undergo an altered differentiated phenotype, normal and LPI monocytes were cultured in the presence of rGM-CSF (10 ng/ml) so as to obtain Monocyte-Derived Macrophages (MDM). Light microscopy revealed that, after rGM-CSF exposure, the majority of LPI MDM (Figure 6, Panel B) exhibited the classical adherent "fried egg" morphology, typical of normal MDM (Figure 6, Panel A). The differentiation into mature macrophages was also evaluated through gene expression analysis. In particular we studied CD204 (Scavenger Receptor A, SR-A), a protein involved in cholesterol uptake [26]; PPARγ (Peroxisome Proliferator-Activated Receptor-γ), a key mediator of surfactant catabolism by alveolar macrophages [27,28]; LPLA2 (Lysosomal PhosphoLipase A2), the enzyme selectively expressed in alveolar macrophages responsible for phospholipid homeostasis [29,30]; and PU.1, a transcription factor that promotes AM maturation, differentiation, and surfactant catabolism [31]. As shown in Figure 6, Panel C, the treatment with rGM-CSF induced a marked expression of all these markers, both in normal and LPI cells, thus demonstrating that LPI monocytes undergo a macrophage differentiation comparable to that of normal cells.

**Figure 7:**
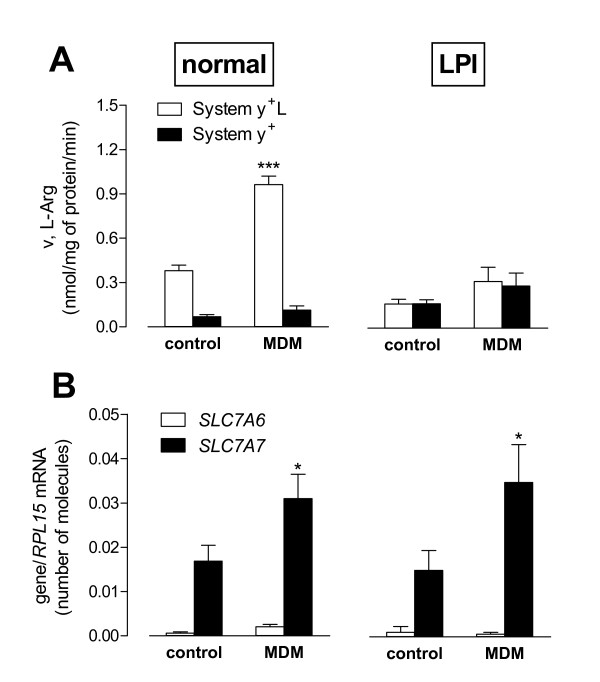
**Effect of GM-CSF on System y**^**+**^**L activity and transporters expression**. Normal and LPI monocytes were differentiated to Monocyte-Derived Marcophages (MDM) as described in Figure 6. Panel A. The activities of system y^+^L and system y^+ ^were determined as described in Figure 3, both in freshly isolated (control) and in rGM-CSF-treated (MDM) monocytes. For normal cells, data are means ± SEM of 6 independent experiments (n = 6 normal donors). For the LPI patient, data are means ± SD of 4 determinations in a representative experiment that was repeated twice with comparable results. ***p < 0.001 MDM vs control. Panel B. In the same conditions *SLC7A6 *and *SLC7A7 *expression were determined as numbers of molecules of mRNA indexed to RPL mRNA (see Methods). For normal cells, data are means ± SEM of 6 independent experiments (n = 6 normal donors). For the LPI patient, data are means ± SD of 4 determinations. * p < 0.05 MDM vs control.

The effects of rGM-CSF on the activity of arginine transport systems (Figure 7, Panel A) were also addressed. A marked stimulation of system y^+^L activity by rGM-CSF was observed in normal but not in LPI monocytes, where the low system y^+^L activity was unaffected by the treatment. In both normal and LPI cells system y^+ ^activity remained substantially unchanged.

In normal cells the stimulation of system y^+^L activity upon incubation with rGM-CSF was fully attributable to the induction of *SLC7A7 *gene (Figure 7, Panel B), thus identifying this gene as a cytokine target. However, also the monocytes from the LPI subject responded to rGM-CSF with a 2-fold increase in *SLC7A7 *mRNA level, although system y^+^L transport activity remained unaffected. Under the same conditions, the expression of *SLC7A6 *did not exhibit any significant change, both in normal and in LPI cells.

## Discussion

To our knowledge, this is the first comparative study on the functional analysis of system y^+^L in cells isolated from different tissues of a patient affected by Lysinuric Protein Intolerance (LPI). We have found that: 1) in AM isolated from the WLL fluid of the LPI patient and in peripheral blood monocytes from the same subject system y^+^L activity is markedly reduced compared to controls; 2) conversely, mesenchymal cells, isolated from the same patient, do not display the transport defect, consistently with our previous results [25]; 3) in both normal and LPI monocytes the expression of *SLC7A7 *is induced by GM-CSF, suggesting that the transporter gene is a target of the cytokine; 4) GM-CSF induces genes coding for specialized macrophage functions in both normal and LPI monocytes.

As far as system y^+^L activity is concerned, the different phenotype displayed by LPI monocytes/macrophages and fibroblasts can be explained by the relative level of expression of *SLC7A7 *and *SLC7A6 *in these models. Indeed, the number of molecules of *SLC7A7 *mRNA in monocytes is markedly higher than that of *SLC7A6 *mRNA, while the opposite is true for mesenchymal cells. Hence, whereas a normal expression of *SLC7A6*/y+LAT2 can compensate for the mutation of *SLC7A7*/y+LAT1 in LPI fibroblasts, the prevailing expression of y+LAT1 in monocytes and AM explains the typical LPI tissue-specific transport defect in these cells.

Operatively, system y^+^L mediates the efflux of cationic amino acids, as demonstrated in both epithelial [32] and non epithelial models [24], including the monocytic cell line THP-1 (results not shown). Because of the genetic defect of *SLC7A7*/y+LAT1, the intracellular concentration of arginine is expected to be higher in LPI monocytes than in normal cells, although the scarce availability of the pathological samples prevents the quantification of arginine content in LPI monocytes/macrophages. Arginine metabolism in myeloid cells is emerging as a crucial determinant of local immune regulation [33]. Indeed, T lymphocytes and NK cells are severely impaired when the extracellular arginine concentration is low [34,35]. Interestingly, clinical outcomes of LPI include immunological abnormalities [36], such as impaired function of lymphocytes, the presence of lupus erythematosus, and an increased susceptibility to haemophagocytic lymphohistocytosis (HLH) [23]. HLH is characterized by an uncontrolled proliferation of CD8+ cells and macrophages, associated with the impaired or absent cytotoxic activity of NK and CTL. Whereas no information is yet available on *SLC7A7 *expression and function in lymphoid populations, the high expression and activity of *SLC7A7 *in normal macrophages and the substantial defect in system y^+^L activity observed in LPI macrophages support the hypothesis of a pathogenetic role of these cells in the development of LPI-associated immunological disorders. Loss of y+LAT1-mediated arginine efflux from LPI macrophages may lead to abnormal arginine availability in the microenvironment, thus affecting CTL and NK functions. The excessive accumulation of intracellular arginine in LPI macrophages may also lead to a deregulated production of nitric oxide (NO) and, in turn, of peroxynitrites (ONOO^-^), which would act as cytotoxic agents for T cells [37]. Although speculative, this hypothesis is consistent with clinical findings showing increased levels of nitrates, the stable metabolites of NO, in the plasma of patients with LPI [38].

Our results demonstrate also, for the first time, that *SLC7A7 *is downstream of GM-CSF signalling and is specifically induced during the maturation of monocytes to macrophages, along with other genes encoding for proteins linked to macrophage functions. GM-CSF induces comparable changes in gene expression also in LPI macrophages, where, however, due to the *SLC7A7 *mutation, no increase in system y^+^L activity is observed. The molecular mechanisms underlying the pathogenesis of autoimmune or hereditary PAP consists of impaired GM-CSF signalling because of neutralizing auto-antibodies to the growth factor or mutations in genes encoding the GM-CSF receptor, respectively [10]. However, we show here that GM-CSF induces comparable morphological phenotypes and expression patterns of differentiation markers in macrophages from LPI patient and healthy subjects. These data demonstrate that the presence of the mutated *SLC7A7 *does not interfere with the GM-CSF-induced macrophage differentiation and indicate that mechanisms other than impaired GM-CSF signalling should be investigated to explain LPI-associated PAP. The involvement of y+LAT1 protein in the catabolism of surfactant also remains to be elucidated.

The progressive refractoriness to WLL and PAP reoccurrence prompted us to consider GM-CSF therapy in our patient, although this approach had been never applied to LPI-associated PAP. On the other hand, administration of the growth factor is a promising therapy for autoimmune PAP, notwithstanding the presence of auto-antibodies anti-GM-CSF [39]. Since our patient was negative for serum anti-GM-CSF autoantibodies [22], GM-CSF would be expected to maintain its biological effects (except the increase in system y^+^L activity). We decided to administer the drug through aerosolization, so as to act particularly on macrophages within the airways and to avoid the troublesome side effects of systemic administration [40]. During the treatment no severe side effects occurred, platelet and red blood cell counts were unchanged and leukocytosis did not develop. Moreover, 12 months after the end of rGM-CSF administration no granulomatous disorder appeared, in contrast with findings by Douda, who had showed that GM-CSF promoted the spontaneous formation of granulomas by LPI alveolar macrophages in vitro [41]. At present, general and respiratory conditions of the patient are good and PAP-related findings have markedly improved. If this positive evolution were to be ascribed to GM-CSF, we should assume that GM-CSF therapy increases also in vivo the expression of genes, other than *SLC7A7*, involved in surfactant catabolism. Alternatively, GM-CSF-dependent increase of *SLC7A7 *mRNA may lead to an enhanced synthesis of y+LAT1 protein that ameliorates macrophage function. This hypothesis, however, implies that *i) *y+LAT1 has also some activities other than arginine efflux; *ii) *the mutations carried by the patient do not interfere with these putative functions and *iii) *y+LAT-1-mediated arginine transport is not directly implicated in surfactant catabolism. Nevertheless, the link between GM-CSF, impaired y^+^LAT1 expression and surfactant accumulation in LPI remains to be elucidated, and our report is not conclusive, taking into consideration the single case and the possibility, although rare, of a spontaneous remission of PAP. Therefore, the establishment of efficacy and safety of rGM-CSF in LPI-associated PAP must await other data.

## Abbreviations used

AM: alveolar macrophages; CAA: cationic amino acids; EBSS: Earle's Balanced Salt Solution; LPI: Lysinuric Protein Intolerance; MDM: Monocyte-Derived Macrophages; PAP: Pulmonary Alveolar Proteinosis.

## Competing interests

The authors declare that they have no competing interests.

## Authors' contributions

Contribution: AB, BMR, RV performed in vitro experiments; BMR and AB isolated the cells; GCG and VD analyzed results; VD and OB designed the research and wrote the paper. GR performed the whole lung lavages, ZK, FM, MLR, and ML performed the clinical assessment of the patient. All authors read and approved the final manuscript.
